# Kinetics and New Mechanism of Azoxystrobin Biodegradation by an *Ochrobactrum anthropi* Strain SH14

**DOI:** 10.3390/microorganisms8050625

**Published:** 2020-04-26

**Authors:** Yanmei Feng, Wenping Zhang, Shimei Pang, Ziqiu Lin, Yuming Zhang, Yaohua Huang, Pankaj Bhatt, Shaohua Chen

**Affiliations:** 1 State Key Laboratory for Conservation and Utilization of Subtropical Agro-bioresources, Guangdong Province Key Laboratory of Microbial Signals and Disease Control, Integrative Microbiology Research Centre, South China Agricultural University, Guangzhou 510642, China; 2 Guangdong Laboratory for Lingnan Modern Agriculture, Guangzhou 510642, China

**Keywords:** azoxystrobin, *Ochrobactrum anthropi* SH14, biodegradation, kinetics, metabolic pathway

## Abstract

Azoxystrobin is one of the most popular strobilurin fungicides, widely used in agricultural fields for decades.Extensive use of azoxystrobin poses a major threat to ecosystems. However, little is known about the kinetics and mechanism of azoxystrobin biodegradation. The present study reports a newly isolated bacterial strain, *Ochrobactrum anthropi* SH14, utilizing azoxystrobin as a sole carbon source, was isolated from contaminated soils. Strain SH14 degraded 86.3% of azoxystrobin (50 μg·mL^−1^) in a mineral salt medium within five days. Maximum specific degradation rate (*q*_max_), half-saturation constant (*K_s_*), and inhibition constant (*K_i_*) were noted as 0.6122 d^−1^, 6.8291 μg·mL^−1^, and 188.4680 μg·mL^−1^, respectively.Conditions for strain SH14 based azoxystrobin degradation were optimized by response surface methodology. Optimum degradation was determined to be 30.2 °C, pH 7.9, and 1.1 × 10^7^ CFU·mL^−1^ of inoculum. Strain SH14 degraded azoxystrobin via a novel metabolic pathway with the formation of *N*-(4,6-dimethoxypyrimidin-2-yl)-acetamide,2-amino-4-(4-chlorophenyl)-3-cyano-5,6-dimethyl-pyridine, and 3-quinolinecarboxylic acid,6,8-difluoro-4-hydroxy-ethyl ester as the main intermediate products, which were further transformed without any persistent accumulative product. This is the first report of azoxystrobin degradation pathway in a microorganism. Strain SH14 also degraded other strobilurin fungicides, including kresoxim-methyl (89.4%), pyraclostrobin (88.5%), trifloxystrobin (78.7%), picoxystrobin (76.6%), and fluoxastrobin (57.2%) by following first-order kinetic model. Bioaugmentation of azoxystrobin-contaminated soils with strain SH14 remarkably enhanced the degradation of azoxystrobin, and its half-life was substantially reduced by 95.7 and 65.6 days in sterile and non-sterile soils, respectively, in comparison with the controls without strain SH14. The study presents *O. anthropi* SH14 for enhanced biodegradation of azoxystrobin and elaborates on the metabolic pathways to eliminate its residual toxicity from the environment.

## 1. Introduction

Strobilurin fungicides represent an outstanding new class of pesticides with higher biological activity and specific modes of action [1,2,3]. Over the last two decades, strobilurin fungicides have been widely used and their applications are steadily increasing [4]. However, extensive application of strobilurin fungicides has raised public concern over environmental contamination and the potential risk to human health [5,6]. Numerous studies have demonstrated that strobilurin fungicides are toxic to both target and non-target species [5,7,8]. Furthermore, strobilurin fungicides might also cause long-term adverse effects on human health [9,10,11,12].

**Figure 1:**
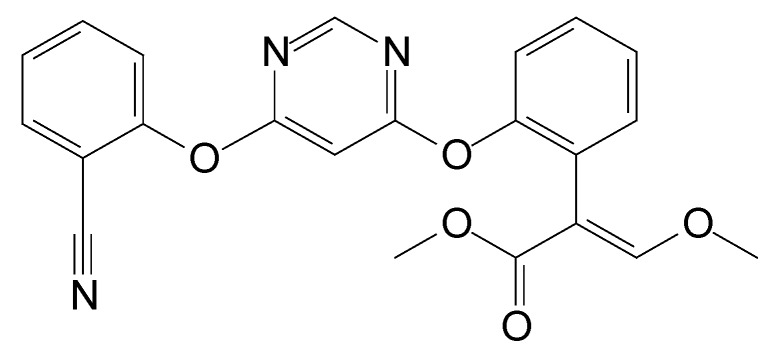
Chemical structure of azoxystrobin.

Azoxystrobin (Figure 1), the first strobilurin compound, was released in 1996 [13]. Despite the fact that azoxystrobin was designed against fungal pathogens with higher curative and control activity, evidence suggest that it may pose a serious threat to the ecosystems [6,14,15]. Berenzen et al. [16] detected azoxystrobin in 12 out of 18 German streams at concentrations of 0.05 to 29.7 μg·L^−1^. According to the European Food Safety Authority, azoxystrobin is one of the most frequently found pesticides in cereal crops and is classified as highly toxic to aquatic organisms or agricultural beneficial organisms [5,17,18,19]. Due to its toxicity towards non-target species, an effective remediation approach for removal of this pesticide is urgently required.

Microbial degradation of pesticide residues has gained popularity as a cost-effective and eco-friendly strategy as compared to conventional methods [20,21,22,23,24,25,26,27,28]. Several strobilurin-degrading strains have been reported such as *Klebsiella* sp. [29], *Stenotrophomonas maltophilia* [30], *Bacillus amyloliquefaciens* [30], *Cupriavidus* sp. [31], and *Rhodanobacter* sp. [31]. However, only a limited number of azoxystrobin-degrading microbes have been explored. Howell et al. [31] reported two bacterial strains (*Cupriavidus* sp. CCH2 and *Rhodanobacter* sp. CCH1) that utilizedazoxystrobin as the sole carbon source for growth. Bacmaga et al. [32] isolated four species of *Bacillus* spp. and two species of *Aphanoascus* spp. from the contaminated soil, which survived up to 22.50 mg·kg^−1^ dose of azoxystrobin. To date, the degradation kinetics and metabolic products of azoxystrobin have not yet been characterized. Furthermore, the degradation mechanism of azoxystrobin has never been investigated and remains unclear.

In this study, an azoxystrobin-degrading strain *Ochrobactrum anthropi* SH14 having superior azoxystrobin degradation activity was isolated, characterized and biodegrading conditions were optimized. Biodegradation assays were also conducted to determine the kinetic parameters. Moreover, the metabolic products were identified and the biochemical degradation pathway of azoxystrobin was proposed. Results elucidated the potential application of *O. anthropi* SH14 for the bioremediation of azoxystrobin-contaminated environments.

## 2. Materials and Methods

### 2.1. Chemicals and Media

Technical-grade azoxystrobin (96.2% purity) was purchased from Wuhan Yuancheng Gongchuang Technology Co., Ltd., Wuhan, China, and chromatographic grade acetonitrile was purchased from Sigma-Aldrich, Shanghai, China. All other chemicals and solvents were of analytical grade. Chemicals were dissolved in acetone to prepare a stock solution of 10,000 μg·mL^−1^ and stored in dark bottles.

Mineral salt medium (MSM) [2.0 g of (NH_4_)_2_SO_4_, 0.2 g of MgSO_4_·7H_2_O, 0.01 g of CaCl_2_·2H_2_O, 0.001 g of FeSO_4_·7H_2_O, 1.5 g of Na_2_HPO_4_·12H_2_O and 1.5 g of KH_2_PO_4_ per liter of water] and Luria–Bertani medium (LB) [5 g of yeast extract, 10 g of tryptone, and 10 g of NaCl per liter of water] were used for the isolation of degraders [33,34]. Both media were adjusted to the final pH of 7.0–7.5 and autoclaved at 121 °C for 20 min prior to use.

### 2.2. Isolation and Identification of Azoxystrobin-Degrading Strains

Approximately 5 g of activated sludge collected from pesticide-manufacturing wastewater treatment system was suspended in 250-mL Erlenmeyer flasks containing 100 mL MSM and 50 μg·mL^−1^ azoxystrobin. The culture was incubated under aerobic conditions at 30 °C and 200 rpm/min on a rotary shaker. After 7 days, 5mL were transferred into another MSM containing 100 μg·mL^−1^ azoxystrobin. After several serial transfers, final cultures were diluted and spread on MSM agar (1.8%) plates containing 50 μg·mL^−1^ azoxystrobin, and incubated at 30 °C for 2 days. Individual colonies were picked out and their azoxystrobin degrading abilities were monitored by high-performance liquid chromatography (HPLC)(Alliance e2690, Waters Corporation, Milford, MA, USA) according to the previous method [35]. A highly efficient azoxystrobin-degrading bacterial strain SH14 was selected and stored in 40% glycerol at −80 °C.

Morphology of strain SH14 was observed on LB agar (1.8%) plates after incubation at 30 °C for 2 days. Genetic analysis was carried out by PCR amplification of 16S rDNA gene with universal primers [36]. PCR cycling conditions were as follows: initial denaturation at 95 °C for 5 min; followed by 32 cycles, denaturation at 94 °C for 45 s, annealing at 50 °C for 45 s, and extension at 72 °C for 75 s and a 10-min extension at 72 °C [37]. PCR products were purified and sequenced from Shanghai Yingjun Technology Co. Ltd., China. Resulting 16S rDNA gene sequence homologies were identified by the BLAST program of the US National Library of Medicine National Institutes of Health (NCBI). Clustal X 1.8.1 was used for multiple sequence alignments with homolog sequences. A phylogenetic tree was constructed following the neighbor-joining method in MEGA 4.0 and strain SH14 was further identified by API 20 NE identification systems (bioMérieux, Marcy l’Etoile, France) [38].

### 2.3. Growth and Biodegradation Experiments

To initiate degradation experiments, strain SH14 was thawed and cultivated on LB agar plates. After 24 h cultivation, the individual colonywas transferred intoLB medium, harvested by centrifugationat 4000× *g* for 4 min, and washed twice with 0.9% sterile saline to prepare a concentrated inoculum solution. Colony-forming units (CFU) of this suspension werequantified by the dilution plate count technique [21,37]. Unless otherwise stated, one percent of this suspension (approximately 1.0 × 10^7^ CFU·mL^−1^) was used as inoculum for all the degradationassays. The suspension was inoculated into 50 mL MSM supplemented with 50 μg·mL^−1^ azoxystrobin as a carbon source and cultivated at 30 °C and 200 rpm for 5 days. Each treatment consisted of three replicates and control cultures were prepared in sterilized MSM with the same concentration of azoxystrobin only. The growth of strain SH14 was determined by counting CFU per milliliter of serial dilutions and concentration of azoxystrobin residues was monitored by HPLC at an interval of 12 h [39].

### 2.4. Optimization of Biodegradation Conditions

Biodegradation conditions of strain SH14 were optimized by response surface methodology (RSM) [40,41,42]. Based on the Central Composite Rotatable Design (CCRD) matrix, three critical factors (temperature, media pH, and inoculum) were designed as the independent variables whereas biodegradation of 50 μg·mL^−1^ azoxystrobin in MSM on day 5 was considered as a dependent variable. Polynomial regression analyses were used to fit the quadratic regression equation (Equation(1)) by response surface regression procedure (REREG) of the statistical analysis system (SAS) software.

(1) $$Y_{i}=b_{0}+\Sigma b_{i}X_{i}+\Sigma b_{\mathrm{ij}}X_{i}X_{j}+\Sigma b_{\mathrm{ii}}X_{i}{}^{2}$$

where *Y*_i_ refers to predicted response(azoxystrobin degradation %), *X*_i_ and *X*_j_ refer to variable parameters. *b*_0_, *b*_i_, *b*_ij_, and *b*_ii_ refer to constant, linear coefficient, interaction coefficient, and quadratic coefficient, respectively.

### 2.5. Biodegradation Kinetics of Azoxystrobin

To test the effect of initial azoxystrobin concentration on its degradation, strain SH14 was inoculated into 250-mL Erlenmeyer flasks containing 50 mL sterilized MSM and different concentrations of azoxystrobin (25, 50, 100, 200, and 400 μg·mL^−1^). Experiments were conducted in triplicate for each treatment, and all the applications were incubated at 30 °C on a rotary shaker (200 rpm) for 5 days. Non-inoculated applications served as controls. Andrews equation (Equation(2)) was followed to determine kinetics parameters of biodegradation at different initial azoxystrobin concentrations [43].

(2) $$q=\frac{q_{max}S}{S+K_{s}+\left(S^{2}/K_{i}\right)}$$

where *S* is azoxystrobin concentration(μg·mL^−1^), *q* is azoxystrobin degradation rate (d^−1^), *q_max_* is maximum azoxystrobin degradation rate (d^−1^), *K_s_* is half-saturation constant (μg·mL^−1^), and *K_i_* is azoxystrobin inhibition constant (μg·mL^−1^). The Andrews equation was applied in Matrix Laboratory software (MATLAB 7.8, MathWorks, Natick, MA, USA).

### 2.6. Biodegradation Kinetics of Various Strobilurin Fungicides

Degradation efficacy of strain SH14 against various strobilurins was studied. Strain SH14 was inoculated into 50 mL sterile MSM containing 50 μg·mL^−1^ azoxystrobin, kresoxim-methyl, pyraclostrobin, trifloxystrobin, picoxystrobin, and fluoxastrobin, respectively. Three replicates were prepared for each treatment and non-inoculated applications served as controls. All cultures were performed in 250-mL Erlenmeyer flasks and cultivated at 30 °C on a rotary shaker (200 rpm) for 5 days. Concentrations of fungicide residues were monitored by HPLC at an interval of 12 h.

The first-order kinetic model (Equation(3)) was created to elucidate fungicides degradation efficiency of strain SH14 [44].

(3) $$C_{t}=C_{0}\times {\;e}^{-kt}$$

where *C*_t_ is the concentration (μg·mL^−1^) of strobilurin fungicides at time *t*, *C*_0_ is the concentration (μg·mL^−1^) of strobilurin fungicides at time zero, and *k* is the degradation rate constant (d^−1^).

Equation (4) was followed to calculate the theoretical half-life (*t*_1/2_) of different strobilurin fungicides.

(4) $$t_{1/2}=\frac{\ln2}{k}$$

where ln 2 is the natural logarithm of 2 and *k* is degradation rate constant (d^−1^).

### 2.7. Identification of Azoxystrobin Metabolites

Strain SH14 was inoculated in MSM containing 50 μg·mL^−1^ azoxystrobin for 5 days in triplicate and cell-free culture filtrates were collected after 12 h. Non-inoculated applications with the same amount of azoxystrobin served as controls. Azoxystrobin metabolites were extracted from cell-filtrates by using ethyl acetate after acidification to pH-2.0 with 2 M HCl [41,45]. The concentrated organic layer was achieved in the rotary evaporator (Heidolph, Schwabach, Germany) and extracts were re-dissolved in acetone. Samples were analyzed by gas chromatography–mass spectrometry (GC-MS).

### 2.8. Biodegradation of Azoxystrobin in Soils

Soil samples were collected from a pesticide-free top 20 cm layer, at a farm in South China Agricultural University, Guangzhou, China. Prior to azoxystrobin application, soil samples were autoclaved for 1 h at 121 °C. The stock solution of azoxystrobin was added to 200 g of sterile and non-sterile soils at a concentration of 50 mg·kg^−1^ [46,47]. Suspension of strain SH14 was introduced into sterile and non-sterile soils at a final bacterial count of approximately 1.0 × 10^8^ CFU·g^−1^ of soil. Control sterile and non-sterile soil received sterile deionized water only. Each treatment was conducted with four replicates. Controls and azoxystrobin amended soils were incubated in a dark thermostatic chamber at 30 °C for 15 days. A 20 g portion of each treatment was aseptically removed at 0, 3, 6, 9, 12, and 15 days to monitor azoxystrobin concentrations.

### 2.9. Analytical Methods

Azoxystrobin concentration was monitored by HPLC equipped with a C_18_ reversed-phase column (Phenomenex, 250 nm × 4.60 mm, 5 μm, Alliance e2695, Waters Corporation, Milford, MA, USA) and a PAD detector at a column temperature of 28 ± 1 °C. Then, 10 μL of each sample was injected and azoxystrobin concentration was determined at 230 nm wavelengths. A mobile phase of 70:30 acetonitrile/water (*v/v*) was used at a flow rate of 1.0 mL·min^−1^ [48].

To identify the azoxystrobin degradation metabolites, extracts were analyzed by GC-MS on a DB-5MS capillary column (30.0 m × 250 μm × 0.25 μm) with an Agilent 6890N/5975 GC-MS system equipped with an auto-sampler and on-column, split/splitless capillary injection system, and an array detection from 40–430 nm (total scan). Operating conditions were as follows: injection volume was 1.0 μL with splitless sampling at 260 °C and helium (>99.999% purity) was used as a carrier gas at a flow rate of 1.0 mL∙min^−1^. Temperatures corresponding to the transfer line and ion trap were 280 °C and 230 °C, respectively, at ionization energy of 70 eV. The column was initially held at 200 °C for 3 min and then raised at 25 °C·min^−1^ to 280 °C for 20 min.

## 3. Results and Discussion

### 3.1. Isolation and Identification of Azoxystrobin-Degrading Strains

Azoxystrobin-degrading bacterial strain SH14 was isolated from the activated sludge that utilized azoxystrobin as a sole source of carbon for growth in MSM. Strain SH14 is an obligate aerobic Gram-negative bacterium that exhibited significantly high azoxystrobin degrading efficiency of 86.3% after 5 days of inoculation. Colonies grown on LB agar plates appeared white, round, convex and circular with a smooth margin. It was positive to glucose, arabinose, maltose, citric acid, and oxidase tests whereas negative in gelatin liquefaction, urea and esculin. Table 1 presents detailed physio-biochemical properties of strain SH14.

**Figure 2:**
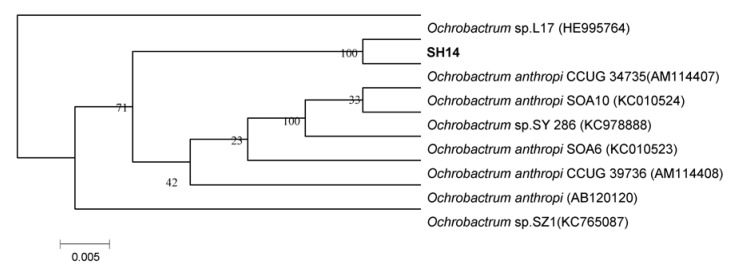
Phylogenetic tree based on 16S rRNA sequence of strain SH14 and related strains. The neighbor-joining method (NJ) was used to construct the phylogenetic tree. Numbers at the nodes show bootstrap values from the neighbor-joining analysis of 1000 resampled data sets. Dates in parentheses are the GenBank sequences accession numbers. Bar refers to sequence divergence.

Based on the phylogenetic analysis of 16S rDNA gene sequences, strain SH14 was tentatively identified as *Ochrobactrum anthropi* and closely clustered with *O. anthropi* CCUG 34,735 (GenBank accession number AM114407) showing over 99% similarity (Figure 2). API 20 NE identification systems classified strain SH14 as *O. anthropi* with 99.9% identification. This isolated strain was deposited in the China Center for Type Culture Collection (collection number: CCTCC M 2013681).

Large-scale applications of azoxystrobin cause several adverse effects on the ecosystem and human health [17,49,50]. Microorganisms provide a cost-effective and eco-friendly solution to pesticide pollutions [34,51,52,53]. However, there is limited information about the microbial degradation of azoxystrobin. In the present study, *O. anthropi* strain SH14 was isolated from azoxystrobin-contaminated soils by enrichment culture technique and it exhibited superb degradation efficiency against azoxystrobin and other strobilurin fungicides.Previous research demonstrated that the bacterial strains from genus *Ochrobactrum* are metabolically active microbes, and they are able to degrade and metabolize various xenobiotics [21,26,27,28]. To our knowledge, this is the first report about azoxystrobin degradation by the *Ochrobactrum* genus of bacteria.

### 3.2. Growth and Utilization of Azoxystrobin by Strain SH14

**Figure 3:**
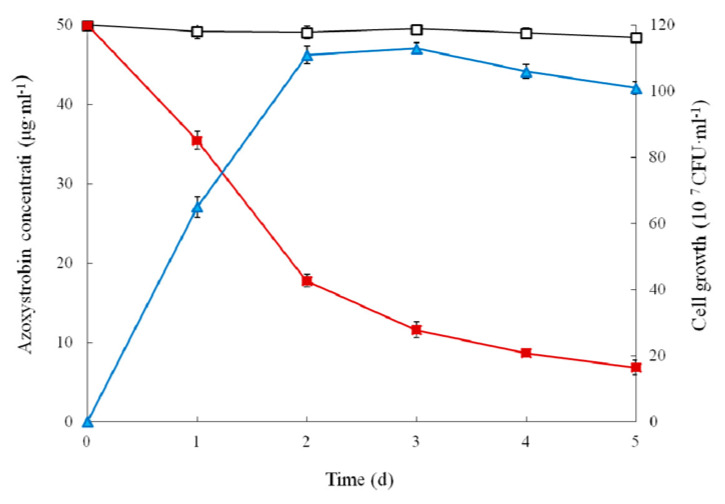
Biodegradation of azoxystrobin (50 μg·mL^−1^) during the growth of strain SH14. Symbol: □, azoxystrobin control; ■, azoxystrobin degradation by strain SH14; ▲, cell growth. Data represent mean values of three replicates with standard deviation.

The dynamic relationship between the growth of strain SH14 in MSM with azoxystrobin as a sole carbon source and its degradation efficiency is shown in Figure 3. Results indicated strain SH14 growth linked degradation of azoxystrobin. Cell numbers of strain SH14 increased to its maximum level followed by a gradual decrease after 3 days of incubation. In comparison to controls, strain SH14 rapidly degraded azoxystrobin by utilizing it as a growth substrate during the exponential and logarithmic phase (0–2 days). After 5 days of incubation, strain SH14 degraded approximately 86.3% azoxystrobin. In the non-inoculated controls, there was no significant change in azoxystrobin concentration and degradation was noted as 3.1%. Reported degradation patterns of azoxystrobin-degrading microbes in the literature are scarce. Previous studies observed that soil microorganisms could grow in the presence of azoxystrobin by utilizing it as a sole carbon source [30,31]. Strain SH14 also consumed azoxystrobin as the sole carbon source that reveals it can be successfully colonized in nutrient-deficient niches.

### 3.3. Optimization of Biodegradation Conditions

Box-Behnken design based response surface methodology (RSM) was used to optimize biodegradation conditions for strain SH14. Temperature (*X*_1_), media pH (*X*_2_), and inoculum (*X*_3_) were designed as three critical independent variables for the enhancement of azoxystrobin biodegradation conditions. Data about the residual amount of azoxystrobin (*Y*_1_) represent the combined effect of these three factors at various levels. The CCRD matrix and response of the dependent variable for azoxystrobin degradation by strain SH14 are presented in Table 2. Quadratic regression equation (Equation(5)) was fitted for the experimental values of azoxystrobin residues, without including those insignificant parameters (*X*_1 × 3_ and *X*_2 × 3_ combinations).

*Y*_1_ = 86.48973 + 1.60726*X*_1_+ 1.830243*X*_2_ + 0.779821*X*_3_ − 11.24968*X*_1_^2^ − 1.075*X*_1_*X*_2_ − 3.259362*X*_2_^2^ − 1.80979*X*_3_^2^ (5)

where *Y*_1_ is the predicted response, *X*_1_ is the temperature_,_ *X*_2_ is the media pH and *X*_3_ is inoculum.

Analysis of variance (ANOVA) of the fitted quadratic polynomial model for azoxystrobin degradation by strain SH14 is listed in Table S1. The coefficient of determination (*R*^2^) value 0.9806 indicates that there is a perfect agreement between predicted and experimental values. Azoxystrobin degradation model reliably represented the relationship between response and independent variables at high significance (*p* < 0.01).

**Figure 4:**
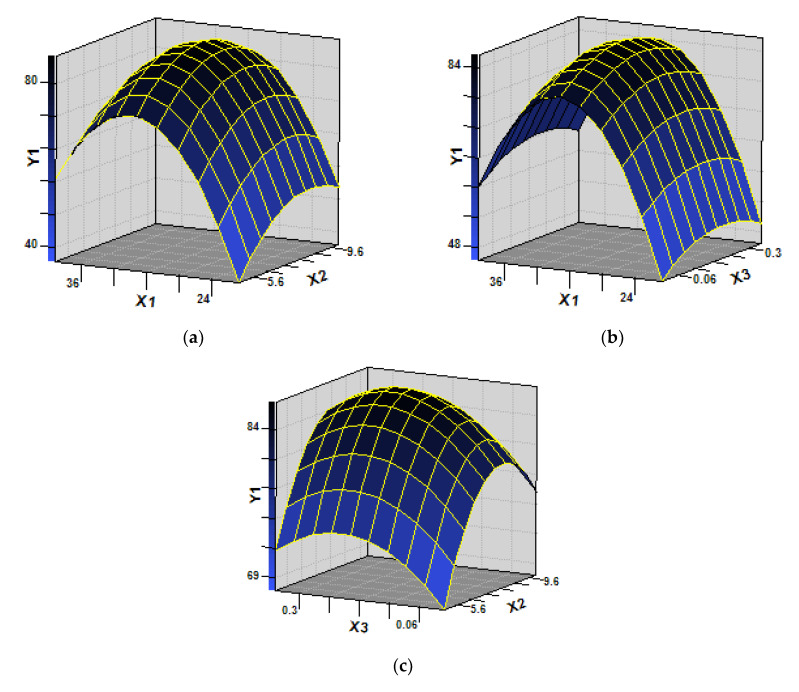
Response surface plots showing the interactive effects on azoxystrobin degradation by strain SH14. (**a**) effect of temperature (*X*_1_) and pH (*X*_2_) on azoxystrobin biodegradation (*Y*_1_) while fixing the value of inoculum size (*X*_3_) at a zero level (1.0 × 10^7^ CFU·mL^−1^); (**b**) effect of temperature (*X*_1_) and inoculum size (*X*_3_) on azoxystrobin biodegradation (*Y*_1_) while fixing the value of pH (*X*_2_) at a zero level (7.5); (**c**) effect of inoculum size (*X*_3_) and pH (*X*_2_) on azoxystrobin biodegradation (*Y*_1_) while fixing the value of temperature (*X*_1_) at a zero level (30 °C).

Single-factor experiments were conducted to determine the effects of important variables such as temperature (*X*_1_), pH (*X*_2_), and inocula(*X*_3_). As shown in Table S1, linear and square terms of temperature (*X*_1_), media pH (*X*_2_), and inocula(*X*_3_) were significant (*p* < 0.05). Three-dimensional response surface plot (Figure 4) intuitively displayed the interactive effect of strain SH14 on azoxystrobin degradation. As shown in Figure 4, the stationary point predicts a maximum value of azoxystrobin degradation. According to the stationary point and quadratic regression equation (Equation(5)), optimum conditions for the three variables (*X*_1_, *X*_2_, and *X*_3_) were determined as temperature 30.2 °C, pH 7.9, and 1.1 × 10^7^ CFU·mL^−1^ of inoculum, respectively.

RSM has been previously used to determine optimum biodegradation conditions in a variety of microbes [42,54,55]. The RSM based quadratic polynomial model (Equation(1)) reliably represented the relationship between degradation effect of strain SH14 and optimum degradation conditions. During this study, a quadratic polynomial model (Equation(5)) was successfully developed to optimize azoxystrobin biodegradation by strain SH14 at temperature 30.2 °C, pH 7.9, and 1.1 × 10^7^ CFU·mL^−1^ of inoculum.

### 3.4. Biodegradation Kinetics of Azoxystrobin

**Figure 5:**
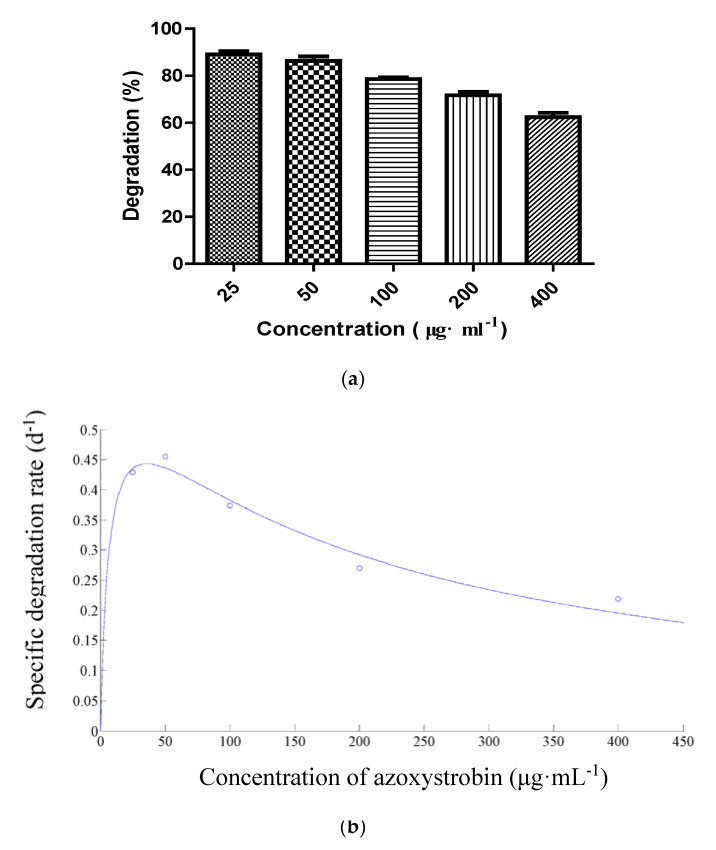
(**a**) Degradation of azoxystrobin at different concentrations; (**b**) Relationship between initial azoxystrobin concentration and specific degradation rate (*q*) of strain SH14. The dot in (**b**) refers to the specific degradation rate (d^−1^) at the concentration of 25, 50, 100, 200, and 400 μg·mL^−1^ of azoxystrobin, respectively.

Strain SH14 was inoculated in the media containing different initial azoxystrobin concentrations (25–400 μg·mL^−1^) to explore azoxystrobin degradation kinetics. As shown in Figure 5a, strain SH14 degraded azoxystrobin up to the concentration of 400 μg·mL^−1^ at degradation of 62.4%. However, some previous studies had reported complete repression of pesticide-degraders at high concentrations [56,57,58]. In this study, a high concentration of azoxystrobin slightly affected the degradation process but complete repression was not observed. It suggests that strain SH14 has a competitive advantage in an adverse environment. At lower azoxystrobin concentrations of 25, 50, 100, and 200 μg·mL^−1^, degradation reached up to 89.1%, 86.3%, 78.5%, and 71.7%, respectively within 5 days. Figure 5b depicts the relationship between different initial azoxystrobin concentrations and a specific degradation rate of strain SH14. We noted that a high concentration of azoxystrobin caused slight repression on the biodegradation process of strain SH14. Results indicated that azoxystrobin degradation by this strain was concentration-dependent. The substrate inhibition model (Equation(2)) adapted from Luong [59] was followed to determine the specific degradation rate (*q*) at different initial azoxystrobin concentrations.

Kinetics parameters *q_max_*, *K_s_*, and *K_i_* of the Andrews equation (Equation(2)) were determined as 0.6122 d^−1^, 6.8291 μg·mL^−1^, and 188.4680 μg·mL^−1^, respectively. The determination coefficient (*R*^2^) of 0.9657 indicated a clear correlation among experimental values and the Andrews equation. Furthermore, a critical inhibitor concentration of azoxystrobin was found as 35.8757 μg·mL^−1^. At azoxystrobin’s initial concentration of lower than 35.8757 μg·mL^−1^, the *q* value was increased rapidly (Figure 5b). At a higher concentration (>35.8757 μg·mL^−1^), inhibition of degradation became prominent. Our results indicated that strain SH14 possesses high azoxystrobin degrading efficiency within the initial concentration of 400 μg·mL^−1^ (*q* > 0.2 d^−1^), highlighting its promising potentials as an ideal microorganism to be employed for bioremediation of variable environments.

### 3.5. Identification of Metabolites

**Figure 6:**
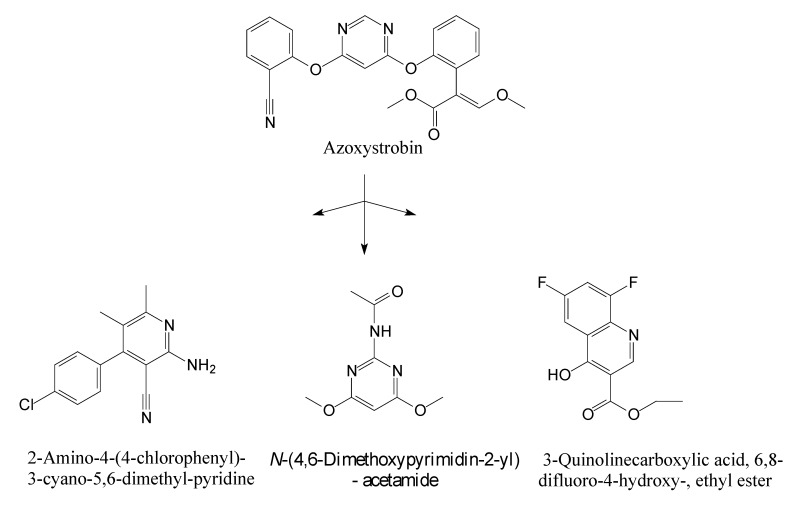
Proposed metabolic degradation pathway of azoxystrobin in strain SH14.

HPLC and GC-MS studies were conducted to monitor the degradation of azoxystrobin by strain SH14. Azoxystrobin intermediate metabolites were extracted and confirmed by GC-MS on the basis of similarity among characteristic fragment ion peaks and authentic standard compounds in the National Institute of Standards and Technology (NIST, USA) library database. Table 3 summarizes the predicted chemical structures, retention times (RT), and characteristic ions of the mass spectra (*m*/*z*). In the sample of 12 h, a significant peak of compound A was detected at an RT of 22.869 min. The protonated molecular ion of this compound at m/z 403 exactly matched with the authentic standard of azoxystrobin in the NIST library database (Figure S1). Furthermore, compound B (9.126 min), C (14.754 min), and D (15.521 min) were detected along with the degradation of azoxystrobin by strain SH14 and were identified as *N*-(4,6-dimethoxypyrimidin-2-yl)-acetamide, 2-amino-4-(4-chlorophenyl)-3-cyano-5,6-dimethyl-pyridine and 3-quinolinecarboxylic acid,6,8-difluoro-4-hydroxy-ethyl ester (Figure S1). At the end of experiment, these metabolites faded away and no cleavable metabolite was detected.The metabolic pathway of azoxystrobin in strain SH14 was proposed by analyzing the chemical structures of azoxystrobin and its metabolites (Figure 6). Hydrolysis of ester linkage and degradation of the aromatic ring in azoxystrobin yielded compounds B, C, and D. This is the first report about the azoxystrobin degradation pathway in a microorganism.

The complex structure of azoxystrobin provides several sites for metabolic reactions by microorganisms [1]. It was generally considered that ester hydrolysis via esterases was the primary pathway of metabolism and detoxification of azoxystrobin in many species, from mammals and plants to microbes [3,60,61,62,63,64]. Similarly, we found that biodegradation of azoxystrobin occurred by the hydrolysis of the carboxyl ester bond, suggesting the central role of esterase in azoxystrobin degradation. What is more, in addition to hydrolysis of carboxylic ester, *O. anthropi* SH14 further degraded azoxystrobin by cleavage of the aromatic ring, leading to complete metabolism and detoxification of azoxystrobin.

### 3.6. Biodegradation Kinetics of Various Strobilurin Fungicides

**Figure 7:**
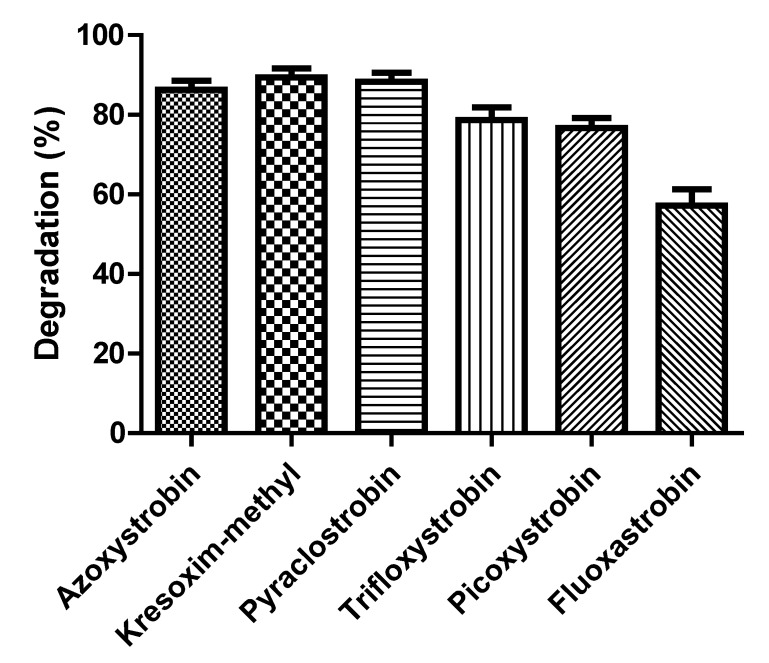
Degradation of various strobilurins by strain SH14 within 5 days. Data represent mean values of three replicates with standard deviation.

The degradation rate of various strobilurins by strain SH14 was detected and explained in Figure 7. Results demonstrated that strain SH14 utilized and degraded all the tested compounds as growth substrates. After 5 days of incubation, degradation of azoxystrobin, kresoxim-methyl, pyraclostrobin, trifloxystrobin, picoxystrobin, and fluoxastrobinwas noted as 86.3%, 89.4%, 88.5%, 78.7%, 76.6%, and 57.2%, respectively. Strain SH14 degraded a wide range of strobilurin fungicides that advocates its promising and competitive role in bioremediation. Similarly, Howell et al. [31] have also reported microbial degradation of azoxystrobin. Microbes degraded different strobilurin fungicides including trifloxystrobin, pyraclostrobin and kresoxim-methyl. However, trifloxystrobin-degraders isolated by Clinton et al. [30] could not degrade azoxystrobin. This distinction can be attributed to the substrate specificity or no-substrate specificity of catabolic enzymes associated with strains. Previous reports about herbicides also had the same observation [27,52,65,66] and another reason could be the similar structure of tested compounds as reported in pyrethroids [67,68,69,70].

The first-order kinetic model (Equation(3)) was created to elucidate the degradation ability of strain SH14 against various strobilurinfungicides. Table 4 presents the kinetics parameters calculated from the model. The coefficient of determination *R*^2^ varied from 0.9419 to 0.9902 indicating that the degradation data reliably fitted with the first-order kinetic model. As shown in Table 4, degradation rate constants (*k*) varied from 0.1857 to 0.5161 d^−1^ that characterized the degradation process of various strobilurin fungicides by strain SH14. Theoretical half-life (*t*_1/2_) of azoxystrobin, kresoxim-methyl, pyraclostrobin, trifloxystrobin, picoxystrobin, and fluoxastrobin was noted as 1.52, 1.34, 1.35, 1.80, 2.06, and 3.73 days, respectively. The *t*_1/2_ for strobilurin fungicides in field conditions often ranges from3.31 to 279 days [5,71,72,73]. Strain SH14 drastically reduced the *t*_1/2_ of various strobilurin fungicides as compared to under field conditions.

### 3.7. Biodegradation of Azoxystrobin in Soils

To investigate the bioremediation potential of strain SH14 in azoxystrobin-contaminated soils, tests were conducted under controlled conditions. The degradation process was further studied with first-order kinetic model and kinetic parameters are tabulated in Table 5. According to the data, the coefficient of determination *R*^2^ varied from 0.9447 to 0.9892 indicating that the experimental data were well fitted with the first-order model. *k* characterized the degradation process and ranged between 0.1857 to 0.5161 d^−1^. *t*_1/2_ of azoxystrobin was calculated as 108.3 and 75.3 d in sterile and non-sterile soils, which drastically reduced to 12.6 and 9.7 days, respectively with the application of strain SH14. These results suggest that strain SH14 is quite potent for the bioremediation of azoxystrobin-contaminated environments.

Previously, low activity of bacterial isolates under field conditions restricted its field applications [74,75,76,77,78]. However, interestingly during this study, strain SH14 quickly adapted to the field conditions without any other treatment. Half-life of azoxystrobin in non-sterilized soils was also significantly shorter than in sterilized soils, indicating that the indigenous soil microorganisms had a synergistic effect on degradation ability of strain SH14.Similar results were observed in previous studies [43,44,79,80,81,82]. *O. anthropi* is a widespread bacterium in natural habitats and has broad catabolic abilities, and it is capable of degrading various xenobiotic compounds including chlorate, cyhalothrin, cypermethrin, etc. [83,84,85]. However, *O. anthropi* has been described as an opportunistic pathogen that may cause infections in immunocompromised persions [86]. Thus, further studies are still needed before the application of the strain SH14 in the field-scale bioremediation, such as the the genetic and toxicological aspects from the bacterial strain. In addition, the azoxystrobin-degrading enzymes and correlated genes in strain SH14 should be explored, which will enable amuch broader application of strain SH14 in azoxystrobin removal.

## 4. Conclusions

In this study, a newly isolated bacterial strain *O. anthropi* SH14 having superior azoxystrobin degradation activity was characterized. Strain SH14 was capable of rapidly degrading azoxystrobin without a lag phase over a broad range of temperature (22 to 38 °C) and pH (5.0 to 10.0). This feature gives the pesticide degrader a competitive advantage in variable environments. It is noteworthy that strain SH14 tolerated and degraded azoxystrobin up to a concentration of 400 mg·L^−1^, thus giving it an exceptional ability to colonize ecological niches where pesticide concentration is high. Strain SH14 transformed azoxystrobin by hydrolysis of the ester linkage and cleavage of the aromatic ring and to yield three intermediates, without any persistent accumulative product, suggesting that this particular strain may harbor a complete metabolic pathway for degradation and detoxification of azoxystrobin. This is the first report of a pathway of degradation of azoxystrobinin a microorganism, which is of vital importance in azoxystrobin biogeocycle. Another important feature which is worth mentioning is that strain SH14 significantly enhanced the removal rate of azoxystrobin in various soils, suggesting a high potentialof applying *O. anthropi* SH14 for bioremediation of an azoxystrobin-contaminated environment. However, further in-depth studies based on genetics and molecular biology of strain SH14 are needed to develop a more safe and efficient strategy to clean up azoxystrobin contamination.

## Figures and Tables

**Table 1:** Physio-biochemical properties of strain SH14 (API 20 NE identification systems).

Characteristics	Results	Characteristics	Results
Glucose	+	Mannitol	−
Arginine	−	Oxidase	+
Arabinose	+	Urea	−
Gelatin	−	Maltose	+
Mannose	+	*N*-acetyl-D-glucosamine	+
Esculin	−	Tryptophan	−
Decanoic acid	+	Glyconate	−
Adipic acid	−	Malic acid	+
Citric acid	+	Phenylacetic acid	−
p-Nitrophenyl β-D-galactopyranoside	−	Potassium nitrate(KNO_3_)	+

Note: +, tested positive; −, tested negative.

**Table 2:** Central composite rotatable design (CCRD) matrix and the response of the dependent variable for azoxystrobin degradation by strain SH14.

Run	*X*_1_	*X*_2_	*X*_3_	Response (*Y*_1_)
				Degradation (%)
1	−1	−1	−1	62.3
2	−1	−1	+1	66.1
3	−1	+1	−1	71.5
4	−1	+1	+1	68.4
5	+1	−1	−1	70.7
6	+1	−1	+1	70.2
7	+1	+1	−1	71.3
8	+1	+1	+1	72.5
9	−1.68	0	0	54.5
10	+1.68	0	0	57.8
11	0	−1.68	0	75.6
12	0	+1.68	0	81.9
13	0	0	−1.68	80.1
14	0	0	+1.68	85.6
15	0	0	0	86.1
16	0	0	0	87.0
17	0	0	0	87.2
18	0	0	0	86.5
19	0	0	0	85.3
20	0	0	0	86.0
21	0	0	0	87.2
22	0	0	0	85.7
23	0	0	0	86.9

Note: *X*_1_ refers to temperature: −1.68 (22 °C), −1 (25 °C), 0 (30 °C), +1 (35 °C), +1.68 (38 °C); *X*_2_ refers to pH: −1.68 (5.0), −1 (6.0), 0 (7.5), +1 (9.0), +1.68 (10.0); *X*_3_ refers to inoculum: −1.68 (0.5 × 10^6^ CFU·mL^−1^), −1 (0.4 × 10^7^ CFU·mL^−1^), 0 (1.0 × 10^7^ CFU·mL^−1^), +1 (1.6 × 10^7^ CFU·mL^−1^), +1.68 (0.2 × 10^8^ CFU·mL^−1^).

**Table 3:** Chromatographic properties of azoxystrobin metabolites during degradation by strain SH14.

Compound	Retention Time (min)	*m/z*	Chemical Structural Formula in NIST Library	Name
A	22.869	403	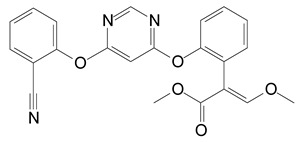	Azoxystrobin
B	9.126	197	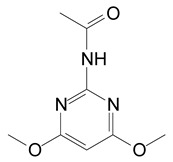	*N*-(4,6-Dimethoxypyrimidin-2-yl)-acetamide
C	14.754	257.5	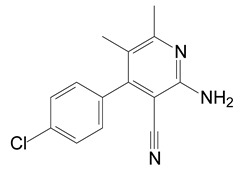	2-Amino-4-(4-chlorophenyl)-3-cyano-5,6-dimethyl-pyridine
D	15.521	253	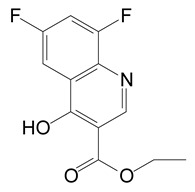	3-Quinolinecarboxylic acid,6,8-difluoro-4-hydroxy-, ethyl ester

**Table 4:** Kinetic parameters of various strobilurin fungicides degradation by strain SH14.

Strobilurins	Regression Equation	*k* (d^−1^)	*t*_1/2_ (d)	*R* ^2^
Azoxystrobin	*C_t_* = 50.8745 × e^−0.4554t^	0.4554	1.52	0.9846
Kresoxim-methyl	*C_t_* = 50.9434 × e^−0.5161*t*^	0.5161	1.34	0.9879
Pyraclostrobin	*C_t_* = 50.3763 × e^−0.5153*t*^	0.5153	1.35	0.9902
Trifloxystrobin	*C_t_* = 51.1746 × e^−0.3845*t*^	0.3845	1.80	0.9626
Picoxystrobin	*C_t_* = 50.9921 × e^−0.3371*t*^	0.3371	2.06	0.9823
Fluoxastrobin	*C_t_* = 53.0503 × e^−0.1857*t*^	0.1857	3.73	0.9419

Note: *k* represents degradation constant (d^−1^); *t*_1/2_ represents half-time (d); *R*^2^ represents determination coefficient; *C*_t_ is the concentration (μg·mL^−1^) of strobilurin fungicides at time *t*.

**Table 5:** Kinetic parameters of azoxystrobin degradation in sterile and non-sterile soils.

Soil Treatments	Regression Equation	*k* (d^−1^)	*t*_1/2_ (d)	*R* ^2^
SS + azoxystrobin	*C_t_* = 19.9387 × e^−0.0064*t*^	0.0064	108.3	0.9447
nSS + azoxystrobin	*C_t_* = 19.9796 × e^−0.0092*t*^	0.0092	75.3	0.9892
SS + azoxystrobin+ SH14	*C_t_* = 20.7399 × e^−0.0550*t*^	0.0550	12.6	0.9824
nSS + azoxystrobin+ SH14	*C_t_* = 20.9485 × e^−0.0715*t*^	0.0715	9.7	0.9597

Note: SS refers to sterile soils; nSS refers to non-sterile soils. *k* represents degradation constant (d^−1^); *t*_1/2_ represents half-time (d); *R*^2^ represents determination coefficient; *C*_t_ is the concentration (μg·mL^−1^) of strobilurin fungicides at time *t*.

## Supplementary Materials

The following are available online at https://www.mdpi.com/2076-2607/8/5/625/s1. Figure S1: Gas chromatography–mass spectrometry (GC-MS) analysis of the metabolites produced during azoxystrobin degradation by strain SH14. (a–d) Characteristic ions of compounds (**A**–**D**). Retention time of each compound was noted as 22.869, 9.126, 14.754 and 15.521 min, respectively and were identified as (a) azoxystrobin; (b) *N*-(4,6-dimethoxypyrimidin-2-yl)-acetamide; (c) 2-amino-4-(4-chlorophenyl)-3-cyano-5,6-dimethyl-pyridine; (d) 3-quinolinecarboxylic acid, 6,8-difluoro-4-hydroxy-, ethyl ester, respectively. Table S1. Analysis of variance (ANOVA) for the fitted quadratic polynomial model of azoxystrobin degradation by strain SH14.
