# Supplementary material for: Kinetics and New Mechanism of Azoxystrobin Biodegradation by an Ochrobactrum anthropi Strain SH14
Source: Microorganisms. 2020 Apr 26;8(5):625. doi: 10.3390/microorganisms8050625 (PMC7284741; doi:10.3390/microorganisms8050625)
Supplement: Supplementary file 1 [file microorganisms-08-00625-s001.pdf]

Kinetics and new mechanism of azoxystrobin biodegradation by an *Ochrobactrum anthropi* strain SH14

Yanmei Feng[1,2], Wenping Zhang[1,2], Shimei Pang[1,2], Ziqiu Lin[1,2], Yuming Zhang[1,2], Yaohua Huang[1,2], Pankaj Bhatt[1,2] and Shaohua Chen[1,2*]

[1]*State Key Laboratory for Conservation and Utilization of Subtropical Agro-bioresources, Guangdong Province Key Laboratory of Microbial Signals and Disease Control, Integrative Microbiology Research Centre, South China Agricultural University, Guangzhou 510642, China*;

[2]*Guangdong Laboratory for Lingnan Modern Agriculture, Guangzhou 510642, China*

[*]Corresponding author

Shaohua Chen

South China Agricultural University, Guangzhou 510642, China

Tel: +86-20-8528 8229

Fax: +86-20-8528 0292

E-mail: shchen@scau.edu.cn

a

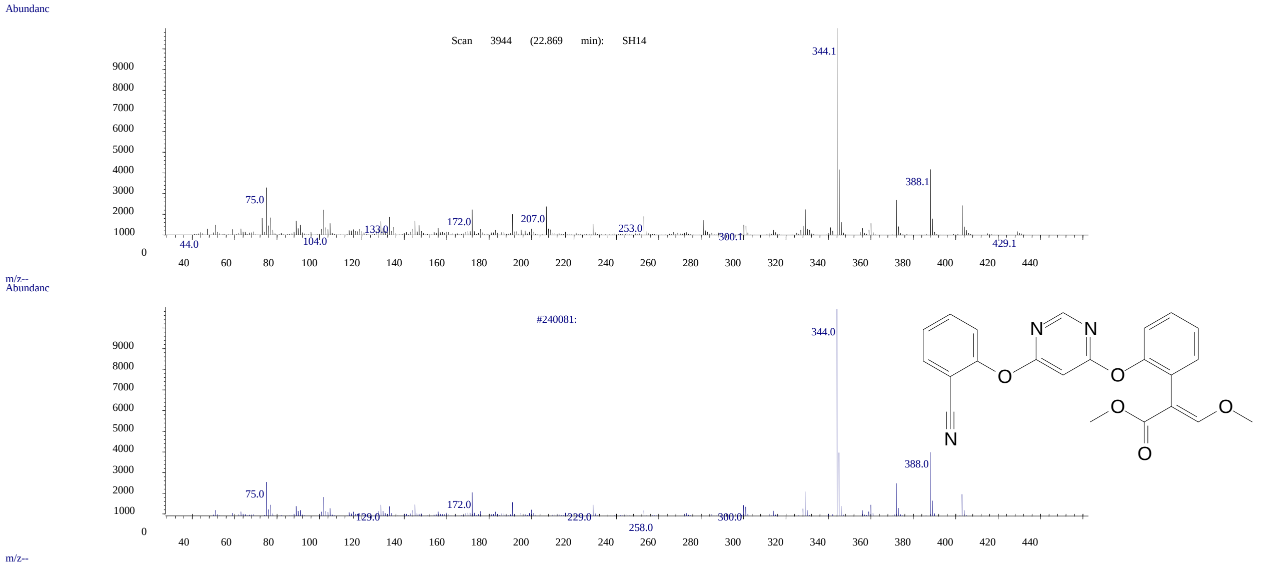
Abundanc
Scan 3944 (22.869 min): SH14
344.1
388.1
75.0
172.0
207.0
133.0
253.0
44.0
104.0
300.1
429.1
m/z--
Abundanc
#240081:
344.0
388.0
75.0
172.0
129.0
229.0
258.0
300.0
N
O
m/z--

b

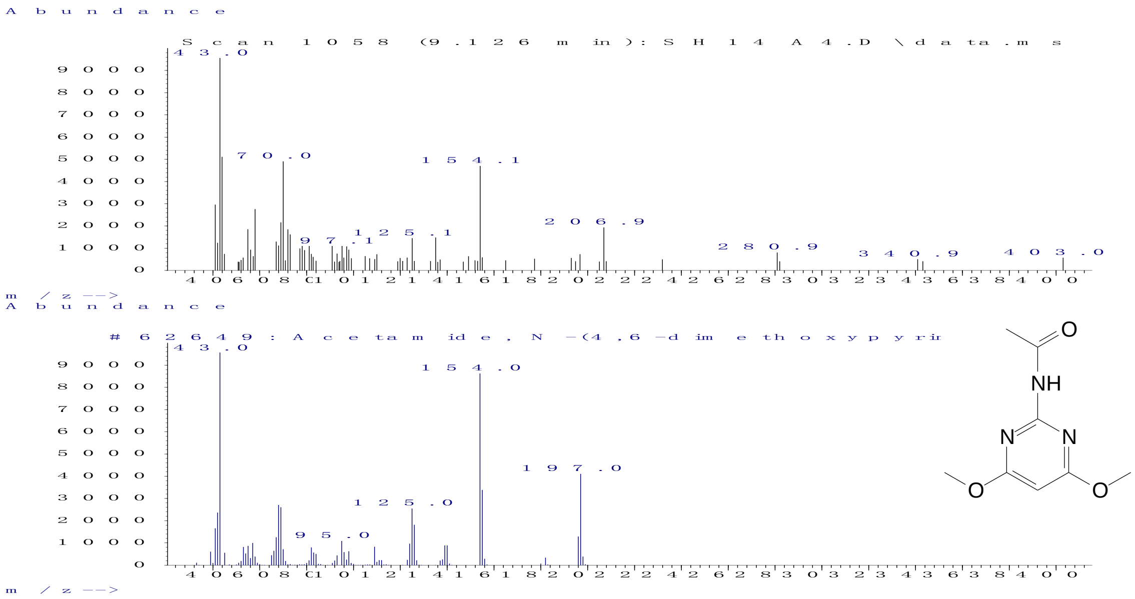
Abundance
Scan 1058 (9.126 min): SH14 A4.D\data.ms
43.0
70.0
154.1
206.9
97.1
125.1
280.9
340.9
403.0
m/z-->
Abundance
#62649: Acetamide, N-(4,6-dimethoxypyri
43.0
154.0
197.0
125.0
95.0
NH
N
O
m/z-->

c

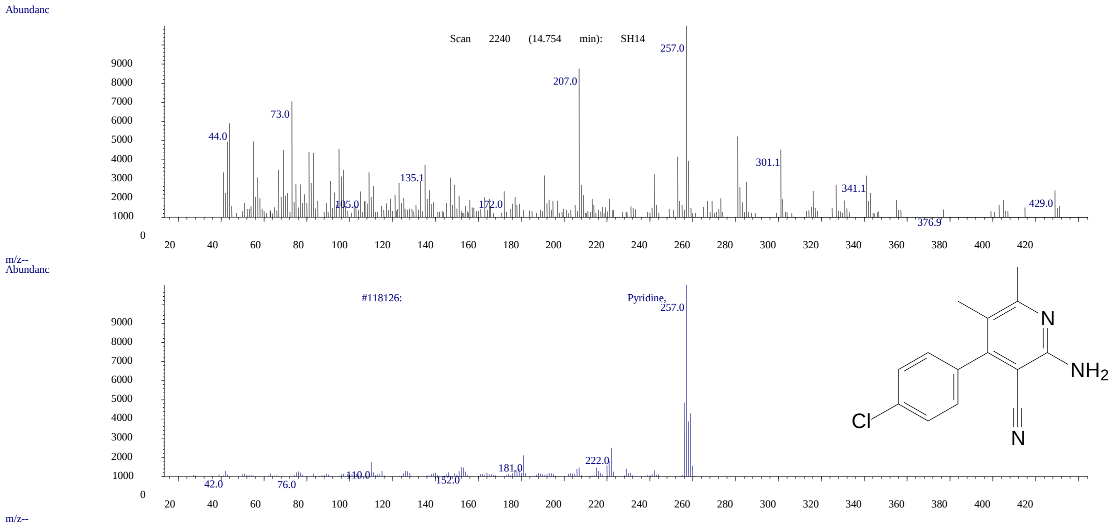

d

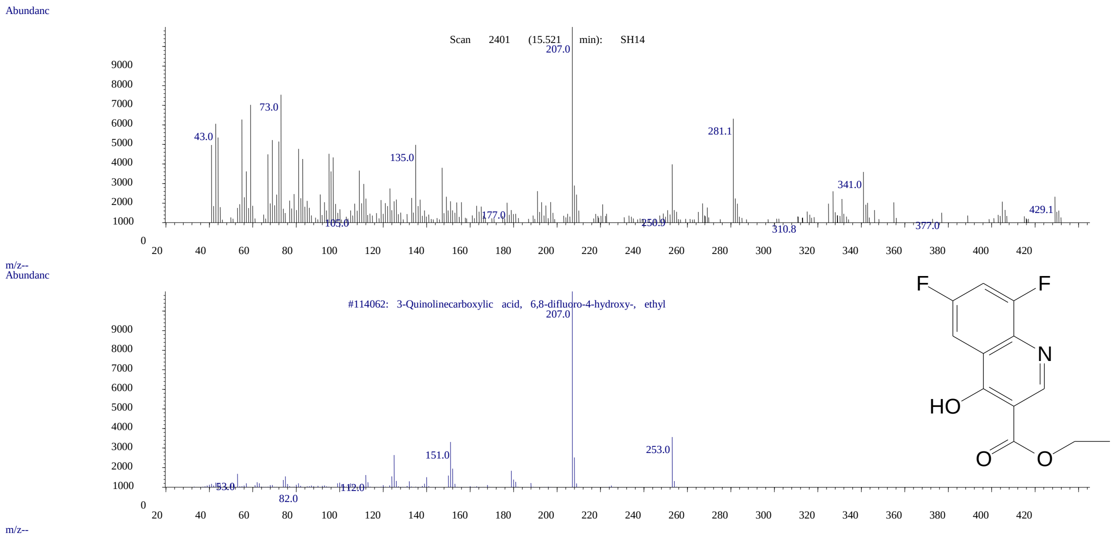

Figure S1. Gas chromatography–mass spectrometry (GC-MS) analysis of the metabolites produced during azoxystrobin degradation by strain SH14. (a–d) Characteristic ions of compounds (A–D). Retention time of each compound was noted as 22.869, 9.126, 14.754 and 15.521 min, respectively and were identified as (a) azoxystrobin; (b) *N*-(4,6-dimethoxypyrimidin-2-yl)-acetamide; (c) 2-amino-4-(4-chlorophenyl)-3-cyano-5,6-dimethyl-pyridine; (d) 3-quinolinecarboxylic acid, 6,8-difluoro-4-hydroxy-, ethyl ester, respectively

Table S1. Analysis of variance (ANOVA) for the fitted quadratic polynomial model of azoxystrobin degradation by strain SH14.

**Source**	**DF**	**SS**	**MS**	***F* Value**	***P* Level** *
X_1	1	35.2795	35.2795	10.0001	0.0074
X_2	1	45.7476	45.7476	12.9681	0.0032
X_3	1	8.305	8.305	2.3542	0.0416
X_1X_1	1	2010.88	2010.88	570.0243	0.0001
X_1X_2	1	9.245	9.245	2.62068	0.0455
X_1X_3	1	0	0	0	1
X_2X_2	1	168.7994	168.7994	47.8496	0.0001
X_2X_3	1	3.38	3.38	0.9581	0.3455
X_3X_3	1	52.0429	52.0429	14.7526	0.002
Model	9	2320.06	257.7955	73.0773	0.0001
Error	13	45.8602	3.5277		
Total	22	2366.02			

Note: R^2= 0.9806, $adj.R^2$= 0.9672. DF refers to degrees of freedom; SS refers to sum of sequences; MS refers to mean square. * *P* Level less than 0.05 indicates that the model terms are significant.
